# Carcinovic cohort: prognostic factors of death in HIV/HCV coinfected patients with hepatocellular carcinoma (HCC)

**DOI:** 10.1186/1471-2334-14-S2-O15

**Published:** 2014-05-23

**Authors:** M Gelu-Simeon, M Lewin, R Sobesky, M Ostos, T Bayan, F Boufassa, L Meyer, A Persoz, E Teicher, H Fontaine, D Salmon-Céron, O Seror, J-C Trinchet, J-C Duclos-Vallée

**Affiliations:** 1 Paul Brousse Hospital, Villejuif, France

## Background and aim

We have previously reported a more advanced radiological presentation in HIV+/HCV+ than HIV-/HCV+ patients (pts). The aim of our study was to define prognostic factors of death in HIV+/HCV+ pts with HCC.

## Methods

Cases of HCC in HIV+/HCV+ pts were obtained from the 3 ANRS Prethevic, HepaVih and CirVir cohorts. Imaging was reviewed according to EASL criteria.

## Results

Fifty HIV+/HCV+ coinfected pts (n=44 men (88%), median age 50 years [40-74], median CD4 cell count 334/mm3 [58-1621], n=28 Child A cirrhosis (60%)) developed HCC. Thirty-one (63%) pts presented cirrhosis decompensation before HCC diagnosis. At HCC diagnosis, median serum aFP was 20.4 [1.9-198,900] ng/ml, 38 (76%) pts had a nodular tumor (median main diameter 23.5 [11-70] cm) and 12 (24%) pts an infiltrating form (62.5 [10-130] cm), p=0.007. Tumor portal thrombosis was diagnosed in 14 (28%) pts. A curative or a palliative procedure was further performed in 22 (44%) pts and 20 (40%) pts, respectively. The 2-years and 4-years overall survival rates were 51% and 28%, respectively. Age (p=0.0005), infiltrating or nodular tumor (p= 0.0009) and tumor portal thrombosis (p=0.004) were associated to survival. In a Cox model, two prognostic factors of deaths were found: prior episode of cirrhosis decompensation (aRR 11.43 [3.01-43.34], p=0.0003) and tumor portal thrombosis (aRR 4.66 [1.19-18.27], p=0.03), adjusted on age, CD4 cell count and the therapeutic strategy for HCC.

## Conclusions

Cirrhosis decompensation and tumor portal thrombosis significantly impact the survival of HIV+/HCV+ pts with HCC. Our results suggest new rules of screening HCC in HIV+/HCV+ pts with advanced liver disorders.

